# Design of Dithiobenzoate RAFT Agent Bearing Hydroxyl Groups and Its Application in RAFT Polymerization for Telechelic Diol Polymers

**DOI:** 10.3390/polym9020044

**Published:** 2017-01-28

**Authors:** Keita Kinoshita, Yuta Mori, Taku Takami, Yusuke Uchida, Yoshihiko Murakami

**Affiliations:** Department of Organic and Polymer Materials Chemistry, Faculty of Engineering, Tokyo University of Agriculture and Technology, 2-24-16 Naka-cho, Koganei, Tokyo 184-8588, Japan; s135908q@st.go.tuat.ac.jp (K.K.); s130383q@st.go.tuat.ac.jp (Y.M.); ttakami@cc.tuat.ac.jp (T.T.); yuchida2@mmm.com (Y.U.)

**Keywords:** RAFT agent, RAFT polymerization, dithiobenzoate

## Abstract

RAFT polymerization is attractive for its reliability, facile operation, and high tolerance to a wide variety of monomers, functional groups, solvents, and temperatures. Herein, we report the RAFT-based synthesis of well-defined polymers bearing hydroxyl groups at two terminals by using various monomers. We found that the molecular weight of obtained polymers was half that of a target value when a trithiocarbonate-type chain transfer agent (CTA) was used, suggesting that the polymers unexpectedly cleaved at the middle of the polymer chain as the reaction was proceeding. To address the problem, we synthesized a novel “dithiobenzoate”-type CTA, 2-[*N*-(2-hydroxyethyl)carbamoyl]prop-2-yl 4-hydroxydithiobenzoate (HECPHD), which bears hydroxyl groups at both terminals, and we succeeded in RAFT polymerization with various monomers without a cleavage of the polymers.

## 1. Introduction

It is important to control the properties of polymers acting as building blocks for the development of functional polymeric materials. The primary structures as well as the assembled higher-order structures of polymers affect the functions of the resulting polymeric materials. Telechelic polymers, macromolecules containing two reactive end groups that can enter into further polymerization or other reactions [1,2], were expected to serve as building blocks for constructing materials. Among versatile telechelic polymers available, hydroxy-functionalized polymers have been widely developed [3,4,5,6], because they are commonly involved in a variety of polycondensation reactions. Of high significance, α,ω-telechelic polymers are quite valuable because they have been used in polyurethanes preparation upon reaction with difunctional isocianates [2]. The reaction between telechelic polymers and multifunctional cross-linkers yields a variety of polymeric networks (hydrogels) that can be used as versatile materials in industrial and medical applications.

Controlled radical polymerization (CRP) is an attractive technique for the preparation of such functional polymers with a precisely controlled molecular structure, a well-defined molecular weight, and a narrow molecular weight distribution. Such techniques that are available to establish living conditions include atom transfer radical polymerization (ATRP) [7,8], reversible addition-fragmentation chain transfer polymerization (RAFT) [9,10], and nitroxide-mediated polymerization (NMP) [11,12]. RAFT polymerization using thiocarbonylthio compounds as chain transfer agents (CTAs) is advantageous for its reliability, facile operation, and high tolerance to a wide variety of monomers, functional groups, solvents, and temperatures. Moreover, RAFT-based synthesis of telechelic polymers is an attractive strategy for designing sequences because the end-group of the polymers depends on the CTA structure. For example, a trithiocarbonate-type CTA, bis[4-(ethyl-2-(hydroxyethyl)aminocarbonyl)-benzyl]trithiocarbonate, which bears hydroxyl groups at both terminals, was used to obtain telechelic hydroxyl polymers and block copolymers for a subsequent successful polyaddition with diisocyanates [13]. It was also reported that *S*,*S*′-bis(2-hydroxyethyl-2′-butyrate) trithiocarbonate was successfully used as a CTA to obtain block copolymers composed of poly(*N*-isopropylacrylamide) and polylactide [14]. Reports on RAFT-based synthesis of telechelic polymers have been limited, although the synthesis of well-defined telechelic polymers acting as synthetic building blocks is a challenging issue that holds promise for the construction of advanced polymeric materials.

**Scheme 1:**
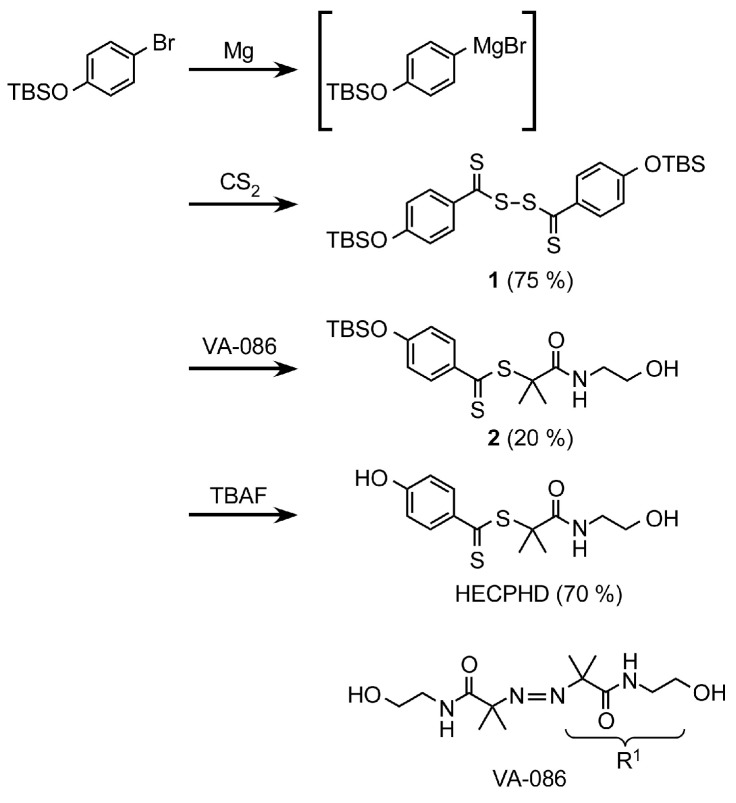
Synthesis of a “dithiocarbonate”-type chain transfer agent (CTA), 2-[*N*-(2-hydroxyethyl)carbamoyl]prop-2-yl 4-hydroxydithiobenzoate (HECPHD), bearing hydroxyl groups at both terminals.

Herein, we report the RAFT-based synthesis of well-defined polymers bearing hydroxyl groups at both terminals using various monomers. First, we attempted RAFT polymerization of acrylamide monomers using a previously reported “trithiocarbonate”-type CTA bearing hydroxyl groups at both terminals [13]. We noted an unusual and interesting phenomenon: the molecular weight of obtained polymers was half that of a target value, suggesting that the polymers cleaved as the reaction was proceeding. To address the problem, we synthesized a novel “dithiobenzoate”-type CTA, 2-[*N*-(2-hydroxyethyl)carbamoyl]prop-2-yl 4-hydroxydithiobenzoate (HECPHD, Scheme 1), which bears hydroxyl groups at both terminals, and we succeeded in RAFT polymerization with various monomers without a cleavage of the polymers. This paper is the first report of RAFT polymerization of various monomers using a “dithiobenzoate”-type CTA bearing hydroxyl groups at both terminals.

## 2. Materials and Methods

### 2.1. Materials

All reagents were purchased from commercial vendors. Styrene (St), ethyl acetate (EA), methyl acrylate (MA), *N,N*-dimethylacrylamide (DMA), *N*-acryloylmorpholine (ACMO), 1,4-dioxane, and chlorobenzene were purified by passing them through basic alumina columns. *N*-isopropylacrylamide (NIPAM) was recrystallized from hexane. Other reagents were used without further purification.

### 2.2. Synthesis of Bis(4-tert-butyldimethylsiloxythiobenzoyl) Disulfide (***1***)

Magnesium (0.607 g, 25 mmol) and tetrahydrofuran (THF, 5 mL) were placed in a nitrogen atmosphere in a 100 mL two-necked round-bottom flask equipped with a reflux condenser. After the addition of an equivalent amount of iodine as a catalyst, THF solution (20 mL) containing 4-bromo(*tert*-butyldimethylsiloxy)phenol (7.18 g, 25 mmol) was added dropwise to the flask. After the contents of the flask were stirred for 3 h, carbon disulfide (1.8 mL, 30 mmol) was added dropwise to the obtained Grignard reagent at 0 °C, and the solution was then stirred for 2 h at room temperature. The solvent was removed under reduced pressure and H_2_O (20 mL) was then added to the residue. After filtration, an aqueous solution (10 mL) of I_2_ (3.17 g, 12.5 mmol) and KI (9.76 g, 58.8 mmol) was added to the filtrate. The resulting dark red precipitates were collected by filtration, washed with water, and dried under reduced pressure (5.34 g, 75%). The obtained product was used without further purification in the reaction discussed in the following section.

### 2.3. Synthesis of 2-[N-(2-Hydroxyethyl)carbamoyl]prop-2-yl 4-tert-butyldimethylsiloxy-dithiobenzoate **2**

Disulfide (**1**) (5.34 g, 9.42 mmol), 2,2′-azobis[2-methyl-*N*-(2-hydroxyethyl)propionamide] (VA-086, 4.07 g, 14.1 mmol), and dioxane (50 mL) were placed in a nitrogen atmosphere in a 100 mL two-necked round-bottom flask equipped with a reflux condenser, and the mixed solvent was heated at 90 °C for 24 h. We obtained compound **2** (0.770 g, 20%) after first removing the solvent under reduced pressure and subsequently purifying the resulting residue by column chromatography with the addition of ethyl acetate as an eluent. The compounds were characterized by ^1^H NMR and ^13^C NMR (ECX-300, 300 MHz, JEOL Ltd., Tokyo, Japan)

^1^H NMR (in CDCl_3_) δ 0.23 (6H, s, C*H*_3_), 0.99 (9H, s, C*H*_3_), 1.77 (6H, s, C*H*_3_), 2.23 (1H, t, *J* = 5.4 Hz, O*H*), 3.38 (2H, m, C*H*_2_), 3.70 (2H, m, C*H*_2_), 6.80 (3H, m, Ar–*H*, N*H*), 8.00 (2H, m, Ar–*H*); ^13^C NMR (in CDCl_3_) δ 4.2, 18.4, 24.6, 25.7, 43.3, 55.8, 62.1, 119.9, 128.9, 138.5, 161.1, 173.7, 223.5.

### 2.4. Synthesis of “Dithiobenzoate”-Type CTA, 2-[N-(2-hydroxyethyl)carbamoyl]prop-2-yl 4-hydroxydithiobenzoate (HECPHD), Bearing Hydroxyl Groups at Both Terminals

A THF solution (1.1 mL) containing 1 M tetra-*n*-butylammonium fluoride (TBAF, 1.1 mmol) was added to the solution containing **2** (0.413 g, 1.0 mmol). After the resulting solution was stirred for 1 h at room temperature, the reaction was stopped by the addition of 0.5 M of aqueous HCl solution. The organic phase after an extraction with dichloromethane was dried with anhydrous Na_2_SO_4_. The mixture was filtered, and the solvent was removed under reduced pressure. The resulting solid that was purified by recrystallization with dichloromethane-hexane yielded HECPHD (0.165 g, 70%).

^1^H NMR (in DMSO-*d*_6_) δ 1.60 (6H, s, C*H*_3_), 3.05 (2H, m, C*H*_2_), 4.53 (1H, t, *J* = 4.1 Hz, O*H*), 6.77 (2H, m, Ar–*H*), 7.64 (1H, t, *J* = 4.5 Hz, N*H*), 7.89 (2H, m, Ar–*H*); ^13^C NMR (in DMSO-*d*_6_) δ 25.7, 42.6, 56.0, 60.1, 115.7, 129.3, 136.7, 163.2, 171.6, 223.4.

### 2.5. RAFT Polymerization of Various Monomers Using CTAs

RAFT polymerization of various monomers was performed by using two CTAs. A typical example of RAFT polymerization using HECPHD is described as follows. Monomers (10.0 mmol), HECPHD (30 mg, 0.10 mmol), and dioxane (10 mL) were placed in a nitrogen atmosphere in a 100 mL two-necked round-bottom flask equipped with a reflux condenser. Afterward, the mixture was degassed in three freeze-pump-thaw cycles, and both VA-086 (7.2 mg, 0.025 mmol) and a few pieces of trioxane were added. Polymerization was carried out at 90 °C for 24 h in a nitrogen atmosphere. After the reaction finished, the mixture was dropped into a 10-fold volume of diethyl ether. The resulting precipitate was collected by filtration and dried under reduced pressure. The polymers finally obtained were characterized by GPC and ^1^H NMR. The conversion of monomers was determined by ^1^H NMR with a comparison between integrated values of monomers and trioxane standard. The molecular weights of the polymers were determined by gel permeation chromatography (GPC, column: TSKgel G3000H_HR_, TOSOH, Tokyo, Japan; eluent: *N,N*-dimethylformamide containing 10 mM LiBr; flow: 1 mL/min; column temperature: 40 °C). Narrowly dispersed polystyrene was used as a GPC standard.

## 3. Results and Discussion

### 3.1. Synthesis of Telechelic Diol Polymers by Means of a Trithiocarbonate-Type CTA Bearing Hydroxyl Groups at Both Terminals

**Scheme 2:**
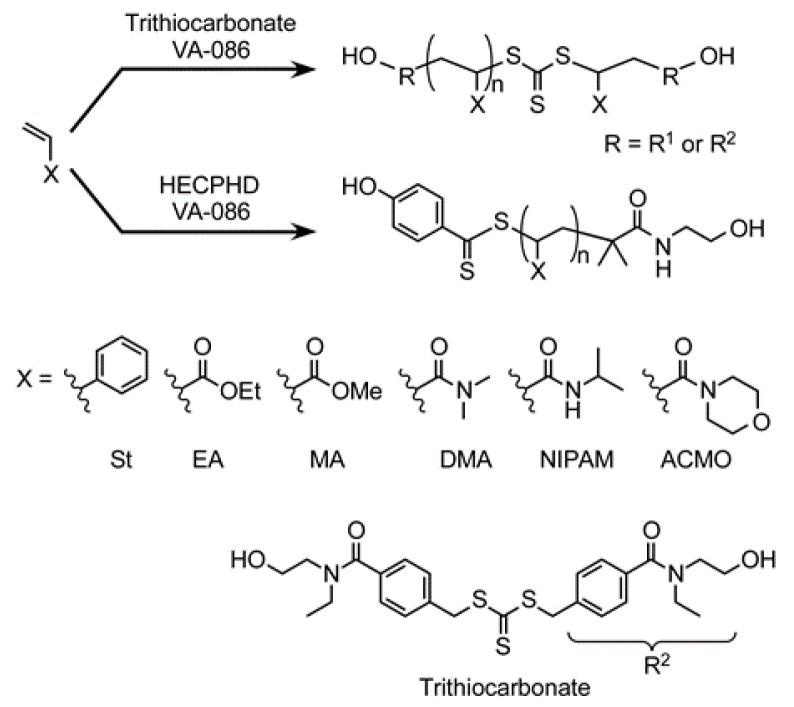
Synthesis of telechelic hydroxyl polymers by trithiocarbonate-type CTA or HECPHD.

We have focused on the preparation of both-terminal hydroxyl-functionalized telechelic polymers by RAFT polymerization. First, as shown in Scheme 2, we obtained telechelic hydoroxyl polymers by a trithiocarbonate-type CTA, bis[4-(ethyl-2-(hydroxyethyl)aminocarbonyl)-benzyl]trithiocarbonate, bearing hydroxyl groups at both terminals. It has been reported that CTAs are useful in the RAFT polymerization of two monomers, St and EA, with predictable molecular weights and narrow polydispersity indexes (PDIs) [13]. DMA, ACMO, and NIPAM have been used as monomers in RAFT polymerization with trithiocarbonate as the CTA (Table 1). Here, an azo compound, 2,2′-azobis[2-methyl-*N*-(2-hydroxyethyl)propionamide] (VA-086), served as an hydrophilic radical source, because the introduction of the compound into the initiating end yields the formed polymer, which bears hydroxyl groups at both terminals. In all the experiments, the initial concentration ratio of monomer to CTA to VA-086 was constant at 100:1:0.25 for synthesizing polymers with a molecular weight higher than 10,000. In all cases, the polymerization proceeded with a monomer conversion over 99% with a low polydispersity index. However, it is noteworthy that, unexpectedly, the molecular weight of the obtained polymer was approximately half that calculated according to monomer conversion.

**Figure 1:**
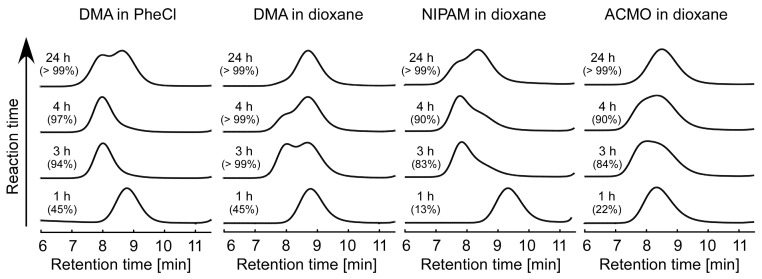
Time course of the RAFT polymerization of DMA, NIPAM, and ACMO.

**Figure 2:**
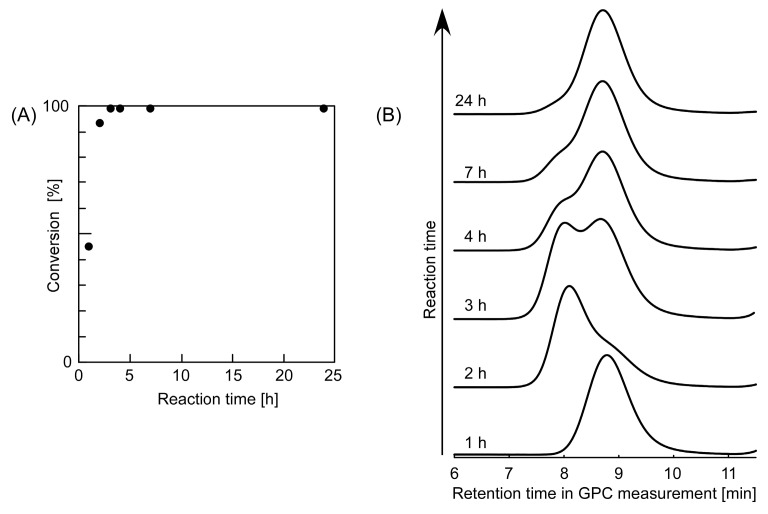
(**A**) Time course of monomer conversions and (**B**) GPC profiles in the RAFT polymerization of DMA in dioxane by using trithiocarbonate-type CTA.

We examined the phenomenon by carefully tracing the time course of the RAFT polymerization by means of a GPC measurement (Figure 1). The appearance and the disappearance of the two types of peaks (i.e., unimodal and bimodal peaks) were observed during RAFT polymerization. Figure 2 shows the detailed time course of monomer conversions and GPC profiles in the RAFT polymerization of DMA in dioxane using trithiocarbonate as a CTA. The monomer conversion drastically increased for 2 h and reached 99% at 3 h. However, the time course of the GPC profiles clearly suggests unexpected behavior; that is, a unimodal peak was separated into bimodal peaks, and another unimodal peak finally appeared as time passed. There were unimodal peaks at 1 h (monomer conversion: 45%) and 2 h (monomer conversion: 93%), whereas, in the latter case, the shoulder peak at the retention time of 9 min gradually appeared. The bimodal peaks appeared at 3 h (monomer conversion: 99%) and gradually evolved into a unimodal peak. These results indicate the cleavage of the chain of the formed polymers. 

It is very likely the case that the cleavage point was a trithiocarbonate unit present at the middle of the polymer chain because the molecular weight of the polymer finally obtained was approximately half that calculated according to monomer conversion. A similar time course of GPC profiles was observed when other acrylamide derivatives, NIPAM and ACMO, were used as monomers. It was generally reported that several phenomena including nucleophilic attacks on thiocarbonyl groups (such as hydrolysis [15] and aminolysis [16]) and thermal decomposition [17,18] induced the degradation of RAFT agents. Furthermore, a trithiocarbonate-type RAFT agent was inactivated by *N*-phenyl-promoted nucleophilic attack of the terminal trithiocarbonate on the ultimate methacrylamide unit in RAFT polymerization of *N*-arylmethacrylamides [19]. Although the precise mechanism for the cleavage phenomena of the polymers obtained in the present study remains unclear, it is noteworthy that the phenomena occurred even when bulky acrylamide derivatives, DMA and ACMO, were used as monomers.

### 3.2. Synthesis of HECPHD

**Figure 3:**
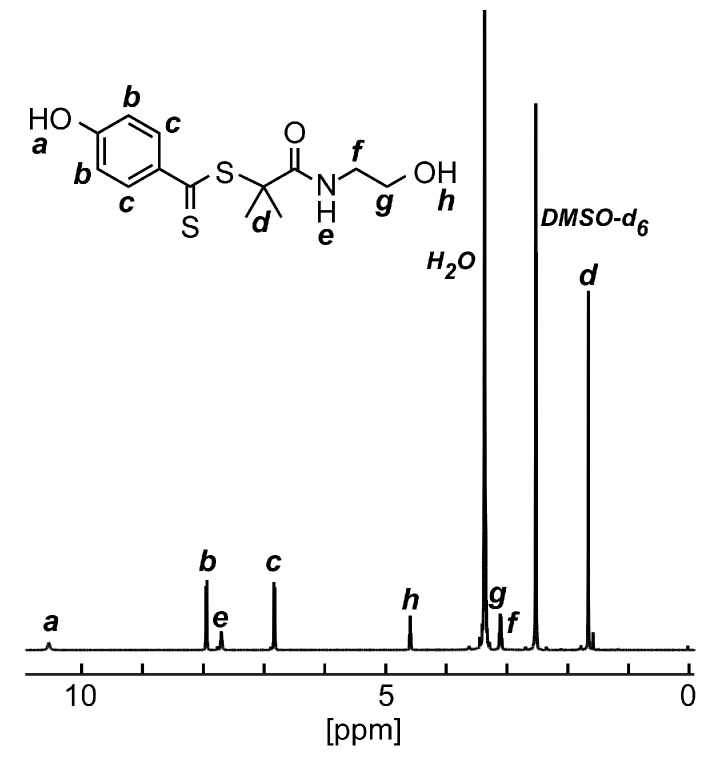
^1^H NMR spectrum of HECPHD in DMSO-*d*_6_.

To address the problems stemming from the unexpected cleavage of polymers, we examined the possibility of developing CTA that can be used for a variety of monomers in RAFT polymerization. As shown in Scheme 1, we synthesized “dithiobenzoate”-type CTA bearing hydroxyl groups at both terminals in three steps. In the first step, we formed a Grignard reagent from TBS-protected p-bromophenol, and the subsequent reaction with carbon disulfide and iodine yielded a significant amount of dithiosulfide (**1**) (75%). In the second step, a radical coupling reaction of dithiosulfide (**1**) with VA-086 yielded dithiobenzoate (**2**) (20%). In the third step, deprotection of **2** with TBAF successfully yielded HECPHD, a CTA bearing hydroxyl groups at both terminals (70%, Figure 3).

### 3.3. RAFT Polymerization of Various Monomers with HECPHD

We investigated the RAFT polymerization of various monomers by using newly synthesized HECPHD as a CTA (Table 2). All reactions were carried out in dioxane at 90 °C for 24 h where the initial concentration ratio of monomer to CTA to VA-086 was constant at 100:1:0.25. The polymerization of EA and MA proceeded with a conversion of approximately 70%, where the PDI was enough to demonstrate the ability of HECPHD to control the RAFT polymerization. In contrast, the polymerization of St proceeded with a low conversion (29%), although the *M*_n_ was the same as the theoretical value, presumably because the high stability of styrene radicals resulted in a slow propagation rate at 90 °C. The low conversion of St has been previously reported: the conversion of St was only 34% in PhCl at 90 °C, whereas it jumped up to 82% at 110 °C [13]. In our case, the conversion of St was improved, reaching 48% at 100 °C (we did not test the reaction in PhCl because of the low solubility of HECPHD in PhCl). The reactions proceeded with high conversions (72%–95%) with relatively low PDI (1.24–1.36) when we used other monomers (MA, DMA, NIPAM, and ACMO).

**Figure 4:**
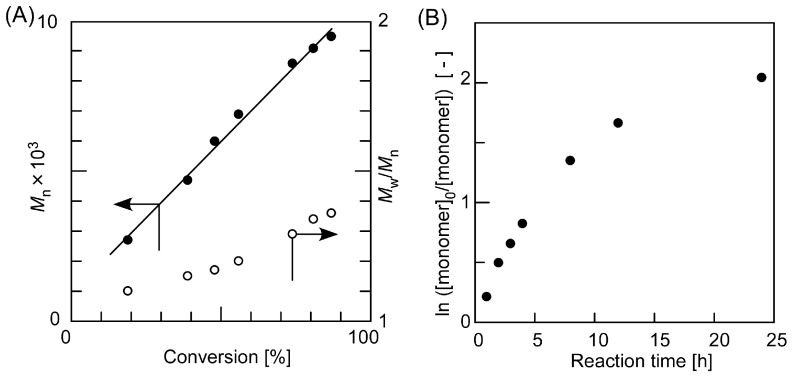
(**A**) The dependence of *M*_n_ and *M*_w_/*M*_n_ on the monomer conversion and (**B**) first-order kinetic plot for the RAFT polymerization of NIPAM.

**Figure 5:**
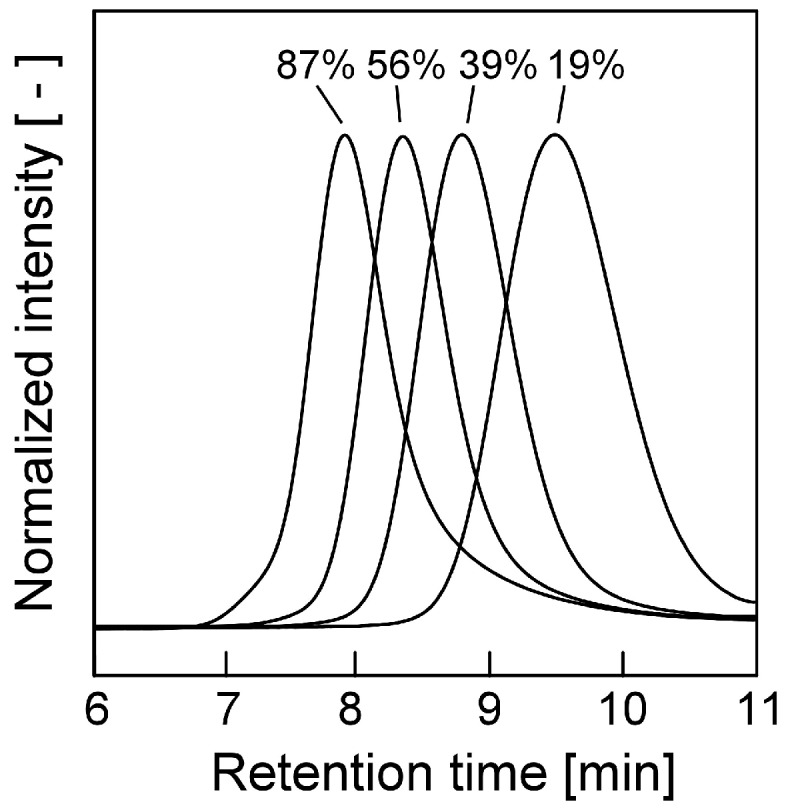
The time course of the GPC profiles of samples in the RAFT polymerization.

**Figure 6:**
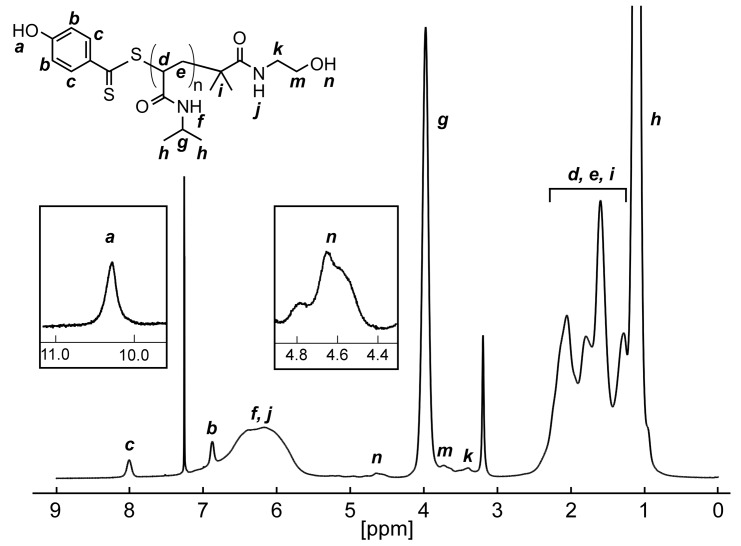
^1^H NMR spectrum of polyNIPAM in CDCl_3_.

**Figure 7:**
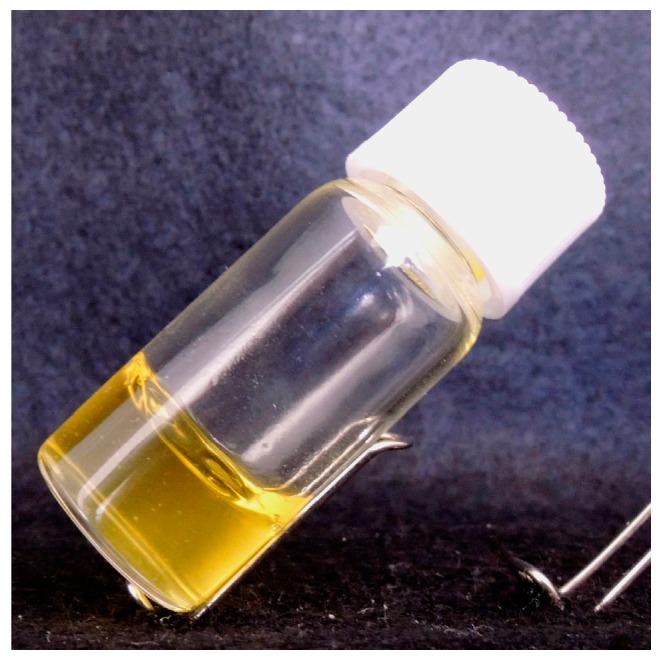
Formation of acrylamide hydrogel by using acyloyled polyNIPAM as a cross-linker ([cross-linker]:[acrylamide]:[APS]:[TEMED] = 2.5:2500:5:50 (molar base)).

Figure 4 shows the typical dependence of *M*_n_ and *M*_w_/*M*_n_ on the monomer conversion and first-order kinetic plot for the RAFT polymerization of NIPAM. A linear increase of *M*_n_ versus conversion was obtained; consequently, the conversion reached 87% with low PDI (1.36). The ln([monomer]_0_/[monomer]) value linearly increased until 8 h (monomer conversion: 74%), indicating that radical concentration persisted regularly until the conversion reached a high value. These results in Figure 4 indicate that RAFT polymerization was well controlled. The time course of the GPC profiles of these samples is shown in Figure 5, suggesting that Gaussian distribution was obtained even at a high conversion and that the emergence of species of compromised chain ends was not observed with a controlled polymerization. Figure 6 shows the ^1^H NMR spectrum of the formed polyNIPAM. The peaks of the terminal dithiobenzoate group were observed at 7.0–8.0 ppm (–C_6_H_4_–, **c** and **b**) and at 10.5 ppm (phenol–OH, **a**). The peaks of the other terminal were observed at 3.5 ppm (**k**, **m**) and 4.5 ppm (**n**). To show the presence of terminal OH groups, we performed an additional experiment. After the acryloylation of the OH groups of the obtained polyNIPAM, the resulting polymers can be used as cross-linkers to form an acrylamide hydrogel (as shown in Figure 7). These results indicated the OH groups at both terminals were present in polyNIPAM molecules, i.e., both incorporation of HECPHD into a polymer chain and RAFT polymerization were successful.

## 5. Conclusions

RAFT polymerization is attractive for its reliability, facile operation, and high tolerance to a wide variety of monomers, functional groups, solvents, and temperatures. Herein, we have reported the RAFT-based synthesis of well-defined polymers bearing hydroxyl groups at both terminals by involving various monomers. We found that the molecular weight of obtained polymers was half that of a target value when a “trithiocarbonate”-type CTA bearing hydroxyl groups at both terminals was used, suggesting that the polymers cleaved as the reaction was proceeding. To address the problem, we synthesized a novel “dithiocarbonate”-type CTA, 2-[*N*-(2-hydroxyethyl)carbamoyl]prop-2-yl 4-hydroxydithiobenzoate (HECPHD), which bears hydroxyl groups at both terminals, and we succeeded in RAFT polymerization with various monomers without cleavage of the polymers. 

The chemical reactivity of the two hydroxyl groups (aliphatic OH and phenolic OH) of the resulting polymers is different when our HECPHD, a dithiobenzoate compound bearing two hydroxyl groups, was used as a RAFT agent. There is a possibility that the difference in the reactivity can be an issue because different reaction conditions are required to modify two hydroxyl groups. Furthermore, there is a possibility that the resulting telechelic diol polymers are unstable because HECPHD (and the corresponding resulting polymers) has a dithiobenzoate structure. These are drawbacks of our RAFT agent, HECPHD. The trial on the further rational design of the dithiobenzoate RAFT agent bearing two hydroxyl groups for the successful synthesis of easy-to-use and stable diol polymers is now underway.

## Figures and Tables

**Table 1:** RAFT polymerization of various monomers with trithiocarbonate-type CTA (concentration of CTA: 0.01 M; reaction time: 24 h; temperature: 90 °C except for St (110 °C)).

Monomers	Solvents	Concentrations [M]		Conversion [%] ^b^	*M*_n,theo_^c^	*M*_n_	*M*_w_/*M*_n_
		Monomers	VA-086				
St ^a^	PhCl	0.5	0.005	82	5000	4400	1.14
EA ^a^	PhCl	0.5	0.005	97	5000	5500	1.15
DMA	PhCl	1	0.0025	>99	10,000	- ^d^	- ^d^
DMA	dioxane	1	0.0025	>99	10,000	5400	1.26
NIPAM	dioxane	1	0.0025	>99	11,300	6000	1.29
ACMO	dioxane	1	0.0025	>99	12,600	6000	1.28

^a^ Conditions and results are from reference [13] for comparison; ^b^ Determined by ^1^H NMR; ^c^ Theoretical *M*_n_ that was calculated according to monomer conversion; ^d^ Bimodal peak was observed.

**Table 2:** RAFT polymerization of various monomers by using HECPHD as CTA. ^a^

Monomers	Conversion [%] ^b^	*M*_n,theo_^c^	*M*_n_	*M*_w_/*M*_n_
St	29	3000	3000	1.12
St ^d^	48	4800	4500	1.13
EA	69	6900	4800	1.33
MA	72	6200	4700	1.24
DMA	90	9000	7200	1.29
NIPAM	87	9800	9500	1.36
ACMO	95	14,000	8300	1.29

^a^ Experimental conditions: The reaction was performed in dioxane at 90 °C for 24 h ([momoners] = 1 M, [CTA] = 0.01 M, [VA-086] = 0.0025 M); ^b^ Determined by ^1^H NMR; ^c^ Theoretical *M*_n_ that was calculated based on monomer conversion; ^d^ Reaction was carried out at 100 °C.
